# Identifying Distinct Profiles of Nutrition Knowledge and Dietary Practices, and Their Determinants Among Adult Women: A Cross-Sectional Study

**DOI:** 10.3390/nu17243916

**Published:** 2025-12-14

**Authors:** Elżbieta Wierzbicka, Barbara Pietruszka, Agata Wawrzyniak

**Affiliations:** Department of Human Nutrition, Institute of Human Nutrition Sciences, Warsaw University of Life Sciences (SGGW-WULS), 02-787 Warsaw, Poland; barbara_pietruszka@sggw.edu.pl (B.P.); agata_wawrzyniak@sggw.edu.pl (A.W.)

**Keywords:** dietary guidelines, nutrition knowledge, eating habits, Poland, adult women

## Abstract

**Background/Objectives:** The purpose of this study was to examine nutrition-related knowledge (NRK) and the implementation of national dietary guidelines (NDGs) as nutrition-related practices (NRPs) among women, identifying profiles and their determinants. **Methods:** A cross-sectional study including 1294 Polish women (mean age 35.8 ± 14.6 years) was conducted in the period June 2021–December 2022 using the Computer Assisted Web Interview (CAWI) method. K-means cluster analysis was applied to standardized variables (NRK and NRP scores, age, and BMI), with the optimal number of clusters determined using the elbow method, to identify major participant profiles representing knowledge and practices with respect to the NDGs. **Results:** Three distinct clusters were identified. The “High” cluster showed median NRK and NRP scores of 15 (IQR = 14–17) and 11 points (IQR = 10–13), significantly higher than those of the “Low” (11 (IQR = 9–13) and 8 points (IQR = 7–10); *p* < 0.001) and “Moderate” cluster (13 (IQR = 11–14) and 7 points (IQR = 6–9); *p* < 0.001), respectively. There were significant differences between clusters in socio-demographics, lifestyle, and health-related distribution. Cluster “High” (38% of sample) had the best NRK and NRP scores and more advantageous socio-economic and lifestyle profiles (higher education, employment, better financial situation, normal BMI, less smoking and higher levels of physical activity), *p* < 0.001; “Moderate” (39%) was characterized by average to relatively good NRK but weaker implementation of NRPs, particularly among younger women, representing more advantageous socio-demographic, lifestyle, and health-related characteristics (*p* < 0.001); and “Low” (23% of sample) comprising middle-aged and older women with lower education, more frequent rural residence, poorer financial status, less favourable lifestyle factors, the lowest NRK and NRP scores, higher prevalence of overweight or obesity, and the lowest level of physical activity (*p* < 0.001)—thus indicating a marked knowledge–practice gap, with this group constituting a potential high-risk population. **Conclusions:** Greater awareness of national dietary guidelines among women is observed alongside higher nutrition-related knowledge and healthier dietary practices. A persistent gap between knowledge and practical implementation among women highlights the need for interventions that support behaviour change alongside nutritional education. Targeted public health strategies are required for women with lower levels of nutrition-related knowledge and less favourable lifestyle profiles.

## 1. Introduction

The importance of nutrition in maintaining health and preventing chronic diseases is well established in the scientific data [1,2]. Many epidemiological studies indicate that unhealthy dietary patterns and insufficient physical activity are now widespread. An unhealthy lifestyle, including inappropriate dietary practices and low levels of physical activity, is associated with an increased risk of adverse health outcomes, including non-communicable diseases (NCDs) such as overweight/obesity, diabetes, cardiovascular disease, hypertension, and others [3,4]. The global increase in the prevalence of overweight and obesity in recent years is particularly concerning [5]. Research conducted in Poland has shown that approximately 53% of women have excessive body weight, and 12.4% are obese [6].

In this context, the most common unhealthy dietary practices among women often include irregular meal routines, skipping meals (e.g., breakfast or dinner), excessively long intervals between meals, and frequent snacking between meals, especially on products containing high amounts of free sugar, saturated fats and/or salt [7,8]. For instance, a repeated cross-sectional survey conducted among women aged 20–50 years living in Poland compared dietary habits in 2011 and 2022. Breakfast consumption was reported more frequently in 2022 than in 2011 (77.6% vs. 63.8%). However, women in 2022 also reported a significantly higher frequency of consuming salty snacks and sweets, highlighting that improvements in selected meal-pattern behaviours may coexist with increases in snacking on energy-dense foods [9]. During the COVID-19 lockdown period in Poland, snacking frequency increased significantly, and the proportion of women snacking several times per day doubled compared with the pre-lockdown period [10]. A further aspect of unhealthy eating is the consumption of ready-made and other highly processed foods, which are recognized sources of simple sugars and salt; Polish studies also indicate excessive contributions of total and saturated fats in high-risk cohorts and high salt intakes, with household and processed foods contributing importantly to dietary sodium [11]. Some women also follow highly restrictive energy intakes or unbalanced weight-loss diets; these patterns may compromise micronutrient adequacy [12]. In addition, a subset of women report emotional or stress-related eating (i.e., eating in response to affect rather than hunger); studies in several contexts have reported higher emotional-eating scores in women than in men, although findings vary by population and setting [13]. Nutrition plays a crucial role in maintaining overall health and overall well-being, so it is important for consumers to have adequate nutrition-related knowledge [14].

Nutrition-related knowledge and practices refer to an individual’s capacity to understand dietary guidelines and translate them into daily eating habits [15,16]. Among women, such knowledge and practices are particularly important, given their specific nutritional needs across the lifespan [17,18]. Significant relationships between nutritional knowledge and the quality of dietary practices have been reported in recent studies [19,20], although these relationships are not always consistently confirmed [21]. Eating behaviors are influenced by many factors; nutritional knowledge may not be sufficient, but it is an essential component of healthy eating patterns [22]. These differences are influenced by intra-individual factors (such as biological, psychological, and personal motivations) as well as broader socio-economic and lifestyle factors [23], in particular physical activity level [24].

Many studies show a significant relationship between nutrition-related knowledge and practice. Consumers with higher levels of nutrition-related knowledge are more likely to make healthier food choices, food preparation, eating habits and diet quality, adhere to dietary guidelines, and consume a more balanced diet [20,25]. Moreover, the findings from other studies suggest that a lack of education can contribute to unhealthy dietary habits, which may lead to an increased risk of chronic diseases such as obesity and cardiovascular disorders [4,17]. In this context, the importance of disseminating dietary guidelines to the general public is emphasized [26]. In many countries, they are developed and considered in various forms, such as a pyramid or a plate [27]. In Poland, guidelines for the general public have also been developed, previously known as the Foods Pyramid [28], and now, since 2020, as the Healthy Eating Plate [29]. Dissemination is primarily nationwide and open-access, via the National Centre for Nutrition Education (NCEZ) website, which provides downloadable guideline graphics for the general public [29].

Despite the availability of national dietary guidelines, evidence remains limited as to how knowledge of these guidelines translates into their day-to-day implementation among adult women, and which subgroups are most likely to exhibit a knowledge-practice gap. While the knowledge–practice gap is recognized [7,8,20,25], there is a lack of studies using cluster analysis to identify risk groups among adult women. Therefore, this study aimed to examine distinct NRK/NRP profiles among Polish adult women and to identify factors linked to potentially high-risk subgroups to enable targeted public health strategies. We hypothesized that nutrition-related knowledge and dietary practices would cluster into distinct profiles among adult women, and that less favourable socio-demographic, lifestyle, and health characteristics would be related to higher-risk profiles, including a pronounced knowledge–practice gap.

## 2. Materials and Methods

### 2.1. Study Design and Participants

A cross-sectional study was conducted in Poland among adult women (≥18 years) using computer-assisted web interviewing (CAWI). Participants completed an online questionnaire via Google Forms© (Online Form Creator). The study was designed to address national principles of healthy eating for the general population, as set out in the Pyramid of Healthy Nutrition and Physical Activity [28] and the Plate of Healthy Eating [29]. These guidelines were developed and endorsed by the National Institute of Public Health—National Institute of Hygiene (NIZP-PZH), in collaboration with the Ministry of Health. To date, similar studies have been published for a younger age group [30].

Participants were recruited between June 2021 and December 2022 through social media (Facebook© (332.0.0.1.111), Instagram© (200.1.0.29.121), and Twitter© (9.29.0)). The inclusion criteria were female sex and age ≥18 years, while pregnancy and lactation were exclusion criteria. The minimal sample size was calculated for a single population using a 95% confidence level, a maximum margin of error of 3%, and 80% power [31]. Assuming that half of women would be aware of the principles underlying the dietary guidelines, the required sample size was 1068 respondents. A total of 1392 women completed the questionnaire; however, due to incomplete or incorrect responses, 1294 questionnaires were deemed eligible for further analysis.

### 2.2. Ethical Considerations

The survey was conducted in accordance with ethical principles. It was anonymous and voluntary. All participants were informed about the purpose and procedures of the study. The study was conducted in accordance with the Declaration of Helsinki and was approved by the Bioethical Commission at the Institute of Human Nutrition Sciences, Warsaw University of Life Sciences (SGGW-WULS), Poland (approval no. 18/2021 as of 1 June 2021).

### 2.3. Measures and Data Collection

A self-administered questionnaire was used to assess nutrition-related knowledge (NRK) and the implementation of dietary guidelines as nutrition-related practices (NRPs). The questionnaire closely aligned with the healthy eating recommendations outlined in the Pyramid of Healthy Eating and Physical Activity for Adults [28] and also incorporated the principles introduced in 2020, presented in the form of the Healthy Eating Plate [29]. The questionnaire was developed in line with expert guidelines of the National Center for Nutrition Education in Poland [28,29]. The response scales and questions were based on the validated Dietary Habits and Nutrition Beliefs Questionnaire (KomPAN^®^), developed by the Committee of Human Nutrition of the Polish Academy of Sciences, which is designed for the Polish population [32]. A pilot study involving 20 individuals from the Faculty of Human Nutrition at WULS was conducted prior to data collection to ensure the relevance and clarity of the questions. Face validity testing was assessed during this phase, and minor refinements to the wording were made accordingly.

Data were collected using a structured questionnaire. It included items on socio-demographic factors, including sex, age, place of residence (village, town with less than 20,000 inhabitants, town with 20,000–100,000 inhabitants, city with more than 100,000 inhabitants), level of education (primary, vocational, secondary, higher), employment (dichotomous choice), self-perceived financial status (very bad, bad, sufficient, good, very good), which were based on the KomPAN questionnaire [32].

Respondents were asked whether they were aware of the national dietary guidelines (NDGs) and lifestyle guidelines as outlined in the Pyramid of Healthy Eating for adults [28], and the Plate of Healthy Eating [29] (dichotomous choice). They were then asked to answer 38 closed questions divided into 2 sections: (1) assessing their nutrition-related knowledge (NRK: 19 questions) and (2) assessing the implementation of dietary guidelines as nutrition-related practices (NRPs: 19 questions), allowing for single-choice response options. The knowledge-related questions also provided the response option “I don’t know”. The question items concerning knowledge and implementation of eating guidelines in practice were equal in number and similar in content. To define correct NRPs, relatively strict criteria that reflected the specific features of the national dietary guidelines as an optimal health-promoting model were applied. This approach was chosen to identify risk profiles and subgroups with the greatest potential for improvement from a public health perspective (Supplementary Tables S1 and S2). In the questionnaire, four distinct subscales were specified. Cronbach’s alpha coefficients were calculated separately for each subscale. The internal consistency of the NRK subscales was satisfactory (Cronbach’s alpha: 0.725, 0.769, 0.782 and 0.835, respectively), and likewise for NRPs (Cronbach’s alpha: 0.716, 0.724, 0.749, and 0.813, respectively). All items were coded in a consistent direction prior to analysis, with reverse-scored items recoded where necessary.

Information on nutritional knowledge and practices was collected from respondents. The questions covered the regularity of meals in daily diet, the selection of foods that should be consumed more frequently: vegetables and fruits; whole-grain cereal products (wholemeal bread, oatmeal, brown rice, wholegrain pasta); fish (particularly fatty sea fish); eggs; low-fat dairy products; and water, and foods that should be limited (red and processed meat; salt; fats; sugar, sweets, and sugar-sweetened non-alcoholic beverages; processed foods; and alcohol). The final question addressed physical activity as a component of a healthy lifestyle (Supplementary Tables S1 and S2).

In addition, the questionnaire included questions on respondents’ self-rated state of health (poor, fair, good, I don’t know), the use of dietary supplements (dichotomous choice), the use of a special diet in the last 6 months (dichotomous choice), self-assessment of physical activity (low, moderate, high), current smoking (dichotomous choice) and alcohol consumption (I do not drink, several times a month, several times a week, several times a day), and self-reported anthropometric measurements (height and weight).

### 2.4. Assessment of NRK and NRPs

The assessment of NRK and the implementation of dietary guidelines as NRPs were based on the points obtained by respondents in the questionnaire. Correct answers were aligned with the healthy eating and lifestyle recommendations for adults in Poland [28,29]. Each correct answer was assigned 1 point, while each incorrect answer received 0 points. Responses of ‘I don’t know’ also received 0 points. The total number of points for both the NRK and NRP scores was 19. The distribution of points for objective NRK results was categorized into four levels: low (0–5 points), moderate (6–10 points), high (11–15) and very high (16–19 points). Similarly, the NRP score was divided into four categories: low—deviating from dietary guidelines (0–5 points), moderate—partially in line with dietary guidelines (6–10 points), high (11–15 points) and very high (16–19 points)—adhering to dietary guidelines.

### 2.5. Assessment of Nutritional Status

The nutritional status of the participants was assessed by body mass index (BMI) calculated from self-reported weight and height. Participants were assigned to categories according to WHO criteria [33]: underweight (BMI < 18.5 kg/m^2^), normal weight (BMI 18.5–24.9 kg/m^2^), overweight (BMI 25.0–29.9 kg/m^2^), and obese (BMI ≥ 30 kg/m^2^).

### 2.6. Statistical Analysis

All statistical analyses were performed using STATISTICA software version 13.3 (TIBCO Software Inc., Palo Alto, CA, USA). The Shapiro–Wilk test was used to assess the normal distribution of parametric data. Continuous variables were presented as means and standard deviations (SD). Since the data showed a skewed distribution, the data were expressed as median, interquartile range (IQR), and range. For continuous or ordinal data, the Wilcoxon signed-rank test was used to compare data from two related (paired) groups, and the Kruskal–Wallis test was used to compare data across three independent groups. Bonferroni’s post hoc correction was applied to multiple comparisons. Categorical variables were presented as numbers (*n*) and percentages (%), and the chi-square test was used to examine differences between categorical variables. The effect sizes were quantified using eta-squared (η^2^) for continuous variables, and Cramér’s coefficient (V) was used for categorical variables.

Cluster analysis based on the K-means algorithm was conducted using NRK and NRP scores, age, and BMI as clustering variables to identify patterns of NRK and NRPs and to examine the relationship with socio-demographic, lifestyle, and health-related characteristics.

All input variables were standardized and expressed as Z-scores, calculated as (value-mean)/SD; a Z-score of 0 represents the mean, positive values indicate above-mean levels, negative values indicate below-mean levels, and the absolute value reflects the distance from the mean in standard deviations (SD). The Elbow method was used to determine the number of clusters in the k-means algorithm (Supplementary Figure S1). The within-cluster sum of squares (WCSS) values, which measure the total distance between each data point and its assigned cluster centroid within each cluster, were plotted to create an elbow point was defined as the optimal number of clusters with support silhouette-score. The overall silhouette score for the final solution was 0.19, indicating that cluster boundaries are relatively indistinct, which may be partly attributable to the influence of external variables. Accordingly, the interpretation was based not only on internal validity indices but also on comparisons across key variables (NRK and NRPs) and their relationships with age and BMI. Three clusters were identified; cluster names (“Low”, “Moderate”, and “High”) were assigned to each of the patterns based on the NRK and NRP Z-score profiles. These patterns were subsequently characterized in relation to socio-demographic and health-related variables.

All reported *p*-values were based on two-sided tests, and the differences were considered statistically significant at *p* < 0.05.

## 3. Results

### 3.1. Characteristics of the Study Population

A total of 1294 women participated in the cross-sectional study. The mean age was 35.8 ± 14.6 years; most women were aged 18–25 (35%), while the remaining age groups (26–35, 36–45, and >45 years) each accounted for 20–24% of the sample. Most women lived in towns (38.0%) or rural areas (35%), had higher (59%) or secondary level of education (29.2%), were employed (68.8%), and reported a good or sufficient financial status (47% and 42%, respectively). Most respondents self-rated their health status as fair and good (37% and 57%, respectively). The majority declared awareness of national dietary guidelines (84%), did not use dietary supplements (61%), and were not following any special diet (86%). Most reported moderate (44%) or low (43.0%) levels of physical activity, were non-smokers (79%) and consumed alcohol infrequently (61%). The majority had a normal body weight (60.7%), while 22.4%, 9.8%, and 7.0% were categorized as overweight, obese, or underweight, respectively. Detailed data on the socio-demographic, lifestyle, and health-related characteristics of the study group are presented in Supplementary Table S1.

Approximately 84% of participants self-reported knowledge of the national dietary guidelines (NDGs), in the form of the Food Pyramid and/or the Healthy Eating Plate [28,29]. As this factor may influence both NRK and the implementation of dietary guidelines as NRPs, further analyses were stratified by this response.

### 3.2. Nutrition-Related Knowledge (NRK)

Women’s responses to questions assessing self-reported NRK in relation to the principles of the national dietary guidelines are presented in Table 1. The data were analyzed in two groups (Yes or No), based on respondents’ declared knowledge of the Food Pyramid and/or the Healthy Eating Plate. Women who reported awareness of the national dietary guidelines generally provided a higher percentage of correct responses across most NRK items compared with those who reported no such knowledge, and these differences were statistically significant (*p* < 0.001). Only two items, relating to the timing of the last meal before bedtime (33% vs. 28%) and the recommended daily water intake (63% vs. 56%), showed no significant differences between the groups (*p* > 0.05) (Table 1).

The NRK items with the highest percentages of correct responses in both groups showed significant differences and were related to limiting sugar-sweetened beverages (96% vs. 87%; *p* < 0.001), choosing healthier alternatives to sweets (94% vs. 83%; *p* < 0.001). Similarly, correct responses were more frequent for reducing intake of high-salt foods (96% vs. 82%; *p* < 0.001), maintaining appropriate breaks between meals (93% vs. 85%; *p* < 0.001).

The greatest difference was observed for the recommended number of daily meals (91% vs. 72%). The lowest accuracy in both groups was recorded for the knowledge regarding the recommended daily intake of dairy products (27% vs. 17%; *p* = 0.003) (Table 1).

### 3.3. Nutrition-Related Practices (NRPs)

Women’s responses to questions assessing self-reported implementation of national dietary guidelines (NDGs) as nutrition-related practices (NRPs) are presented in Table 2. The percentage of correct responses varied markedly, ranging from 7% for the recommended frequency of fish consumption to 78% for the number of daily fruit servings. Statistically significant differences between women with and without awareness of the NDGs were observed for seven items.

Higher proportions of correct responses were noted for meal pattern–related items, including maintaining appropriate intervals between meals (79% vs. 71%; *p* = 0.004), and the number of meals per day (55% vs. 34%; *p* < 0.001).

For food choice–related items, higher accuracy was found for fruit intake (80% vs. 73%; *p* = 0.031), meat selection (72% vs. 59%; *p* < 0.001), and choosing cereal products (43% vs. 24%; *p* < 0.001). For beverage-related items, correct responses for limiting sugar-sweetened beverages (26% vs. 17%; *p* = 0068). The least frequently implemented recommendations concerned important food groups and limiting practices, i.e., fish consumption (8%), vegetable intake (11%), milk and dairy consumption (15%), limiting fast-food intake (16%), and reducing alcohol consumption (19%); no significant between-group differences were observed (*p* > 0.05). Adherence to physical activity recommendations was low in both groups, with only 10% of women meeting the guideline (*p* > 0.05) (Table 2).

### 3.4. Assessment of NRK and NRPs in Relation to Self-Reported Knowledge of National Dietary Guidelines (NDGs)

Table 3 presents the differences in nutrition-related knowledge (NRK) and nutrition-related practices (NRPs) according to self-reported awareness of the national dietary guidelines (NDGs). Median NRK and NRP scores were significantly higher among women who reported awareness of the NDGs (14 and 9 points, respectively) compared with those without such knowledge (10 and 7 points, respectively). In both groups, NRK scores were higher than NRP scores (*p* < 0.001). Among women aware of the NDGs, most demonstrated high or very high NRK (59% and 29%), whereas those without this awareness predominantly showed moderate levels (56%). A similar pattern was observed for NRPs: moderate levels predominated in both groups (59% and 75%, respectively), while high levels were more frequent among women aware of the NDGs (34% vs. 16%); *p* < 0.001 in both.

### 3.5. Profiles of NRK and NRP Cluster Analysis

**Figure 1:**
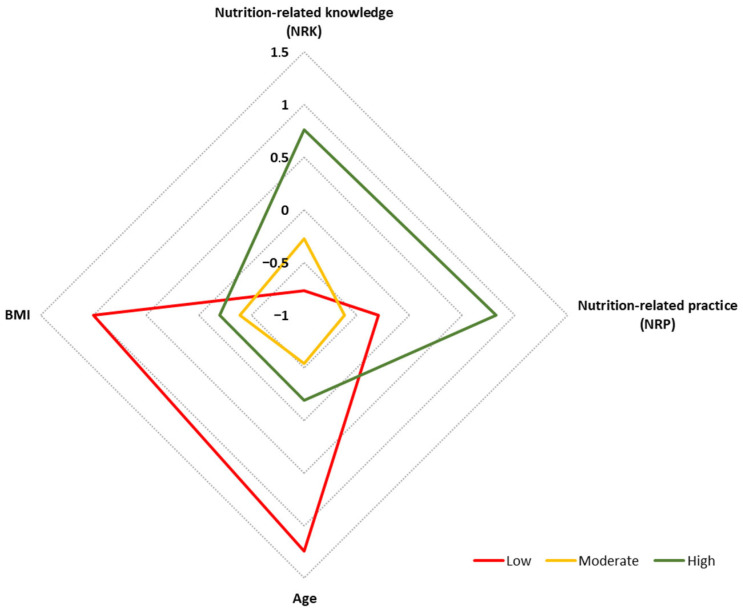
NRK and NRP profiles, including age and BMI, identified using K-means cluster analysis and categorized into “Low” (*n* = 299), “Moderate” (*n* = 503), and “High” (*n* = 492) knowledge of national dietary guidelines. All input variables were standardized and expressed as Z-scores; Z = 0 equals the study mean; positive/negative values indicate above/below mean, and |Z| shows the distance in standard deviations (SD); *n* = 1294.

Cluster analysis identified three subgroups (clusters) of individuals with similar patterns of NRK and NRP scores, incorporating factors such as age and BMI. These clusters were classified as representing “Low”, “Moderate”, and “High” knowledge of national dietary guidelines (Figure 1).

Three clusters were identified based on patterns of nutrition-related knowledge (NRK) and nutrition-related practices (NRPs), together with age and BMI (Table 4). The “Moderate” and “High” clusters were of similar size (39% and 38%, respectively), whereas the “Low” cluster was the smallest (23%). NRK and NRP scores differed significantly across clusters (*p* < 0.001). The “High” cluster demonstrated the most favorable profile, with the highest median NRK (15 points) and NRP (11 points) scores. In contrast, the “Low” cluster showed the lowest NRK (11 points) and a lower-range NRP score (median 8 points), while the “Moderate” cluster exhibited intermediate NRK (13 points) and the lowest NRP score (7 points); significant between-group differences were observed, *p* < 0.001 (Table 4).

Significant differences in age were also observed across clusters (Table 4). Women in the “Moderate” cluster were the youngest (median age 25 years), followed by those in the “High” cluster (30 years), whereas the “Low” cluster comprised the oldest participants (56 years); *p* < 0.001. Although BMI values were similar in “Moderate” and “High” clusters (21.1 and 22.5 kg/m^2^, respectively; *p* > 0.05), the “Low” cluster showed a significantly higher BMI (27.9 kg/m^2^). This cluster was also characterized by a high percentage of women aged over 45 years (68%) and a higher prevalence of overweight (43.8%) or obesity (31.1%); *p* < 0.001 (Table 4).

### 3.6. NRK and NRP Profiles According to Socio-Demographic, Lifestyle, and Health-Related Factors by Clusters

To further characterize the clusters, socio-demographic, lifestyle, and health-related factors were examined. As shown in Table 5, the three clusters differed significantly across these factors. Participants in the “Low” cluster were more likely to live in rural areas (33.8%) and less frequently in cities (27.4%) than those in “Moderate” and “High” clusters (*p* < 0.001). They also had a lower level of education, with a higher prevalence of primary and vocational education (32%) and fewer women with higher education compared with the other clusters (*p* < 0.001). Employment was most common in the “High” cluster (82%), followed by the “Moderate” (69%) and “Low” clusters (63%); *p* < 0.001. A similar pattern was observed for financial status; women in the “High” cluster were more likely to report their financial status as good (57%) or sufficient (34%) than those in the other clusters (*p* < 0.001).

Lifestyle characteristics also varied across clusters (Table 5). Low physical activity was most common in “Low” and “Moderate” clusters (53% and 45%, respectively) and least common in “High” cluster (35%); *p* < 0.001. Use of dietary supplements was less frequent in “Low” and “Moderate” clusters (34% and 32%, respectively) than in the “High” cluster (49%); *p* < 0.001. Smoking was more prevalent in the former two clusters (27% and 26%, respectively) compared with the “High” cluster (14%). Abstinence from alcohol was least common in the “Moderate” cluster (12%), whereas most women in all clusters reported drinking alcohol one to several times per month. Self-rated health also improved progressively across clusters, with 37%, 56%, and 69% of women in “Low,” “Moderate,” and “High” clusters (*p* < 0.001), respectively, rating their health as good (Table 5).

The results highlight considerable variation in nutrition-related knowledge (NRK) and nutrition-related practices (NRPs) among women, with consistent evidence that knowledge exceeded practical implementation. Awareness of the National Dietary Guidelines (NDGs) was linked to higher NRK and NRP scores, although adherence to recommended behaviors remained generally limited. Cluster analysis identified three groups differing in knowledge, practices, age, BMI, and socio-demographic profiles. Women in the “High” cluster (38% of participants) demonstrated the most favorable patterns of nutritional knowledge and behaviour, as well as more favorable lifestyle, health, and socioeconomic characteristics, whereas the “Low” cluster (23%) was characterized by lower knowledge, poorer dietary practices, and less favorable lifestyle factors and poorer self-rated health status.

## 4. Discussion

In this cross-sectional study, we examined self-reported awareness of national dietary guidelines (NDGs), nutrition-related knowledge (NRK), and the implementation of dietary guidelines as nutrition-related practices (NRPs) among Polish women (*n* = 1294), with a mean age of 35.8 ± 14.6 years, as well as their relationship with socio-demographic, lifestyle, and health-related factors. Three NDG-related patterns of nutrition knowledge (clusters) were identified in this population, forming “Low”, “Moderate”, and “High” clusters. To the best of our knowledge, this is the first study to use cluster analysis to explore the relationships between general NRK and NRPs and the corresponding socio-demographic, lifestyle, and health-related factors among adult women in Poland.

In this study, we found that women who reported awareness of national dietary guidelines consistently demonstrated higher NRK and NRP scores than those who did not. They were more likely to provide correct responses regarding, for example, the number of fruit servings per day, intervals between meals, the number of meals per day, and the choice of meat, fats, salty foods, and sweetened beverages. However, even within this subgroup, adherence to several key recommendations remained low, particularly in relation to fish, vegetable, milk, and dairy consumption. A similar pattern was observed for physical activity, where adherence to recommended levels was low across all subgroups, irrespective of awareness of lifestyle guidelines.

This pattern reflects findings from Polish studies showing that higher nutrition-related knowledge is related to associated with better dietary quality, but that knowledge alone is not sufficient to ensure full adherence [25,34,35]. This observation is supported by international data: a systematic review conducted among adults found positive, although weak, relationships between nutrition-related knowledge and food consumption, most often higher fruit and vegetable consumption [36].

In young Polish populations, higher nutrition-related knowledge is linked to healthier eating patterns and higher consumption of health-promoting products, yet significant gaps remain in the consumption of vegetables, fruits, whole grains, and fish [30,37]. Similar unhealthy dietary practices are reported globally, and adherence to national food-based dietary guidelines has been shown to be suboptimal across both high-income and low- and middle-income settings [26]. Similarly, research conducted among women in other countries has shown that although women often perceive themselves as well informed, objective assessments reveal significant gaps in understanding and discrepancies between knowledge and dietary practice [18,38], including data mismatches between perceived and assessed nutrition knowledge and/or guideline-consistent eating habits. For example, data from Switzerland [18] and Italy [39] indicate that many adults perceive themselves as well-informed, yet objective assessments show only moderate knowledge accuracy, limited agreement between perceived and assessed knowledge, and relatively few individuals report dietary habits consistent with their knowledge level [18,39].

When considering the implementation of dietary guidelines (NRGs), we observed that women tended to follow those recommendations that were relatively easy to integrate into existing routines, such as maintaining regular breaks between meals, consuming several meals per day, or choosing lean meat, poultry, and healthy fats. In contrast, more demanding recommendations, including regular consumption of vegetables and fish, limiting fast food, and maintaining sufficient levels of physical activity, were adhered to by only a minority. This pattern is consistent with national data indicating that the overall diet quality of the adult Polish population is typically poor, with particularly low intakes of vegetables, fruits, whole grains, milk, dairy products, and fish [7].

Notably, in our study we found significant gaps in both NRK and NRPs regarding milk and dairy products. The recommended daily intake of milk and dairy products was one of the least correctly identified items in the NRK, and only a small percentage of women who reported knowledge of the guidelines implemented this recommendation in practice. This pattern is consistent with other Polish studies showing that although dairy products are commonly perceived as health-promoting, only a small percentage of women meet the recommended daily intake [40]. In Europe, dietary guidelines recommend daily consumption of dairy products, and many of them provide detailed information about these foods. Despite this, consumption of milk and dairy products remains low in many countries [41], with Poland reporting particularly low dairy consumption rates in comparative analyses [40]. Other studies indicate that consumers often overestimate their knowledge of dairy products, and objective assessments reveal confusion about recommended amounts and types of dairy products [42]. At the same time, dairy recommendations in Europe are increasingly debated, particularly in relation to lactose intolerance, cultural preferences and the growing use of fortified plant-based alternatives as partial substitutes for dairy in some national guidelines [43].

Our findings also reflect global dietary patterns, showing that diet quality remains unhealthy in many countries and that improvements are weakest for key food groups such as whole grains, vegetables, and dairy products, especially among younger adults and those with lower education [44]. By linking these patterns of challenging dietary behaviours, particularly vegetable, fish, and dairy intake, to guideline awareness, we strengthen the argument that the pathway from knowledge to practice is constrained not only by information but also by environmental, economic, and behavioural factors [23,45].

Our cluster analysis adds important interpretative insight to these findings. We identified a “High” cluster in which women (38%) exhibited the most favourable patterns of NRK and NRPs and, simultaneously, more advantageous socio-economic and lifestyle profiles (higher education, employment, better financial situation, a higher prevalence of normal BMI, a lower prevalence of smoking, and higher physical activity). In contrast, the “Low” cluster comprised women (23%) with lower education levels, more frequent rural residence, poorer financial status, less favourable lifestyle factors, and the lowest levels of both NRK and NRPs. The “Moderate” cluster (39%) was particularly noteworthy, as women in this group demonstrated average to relatively good NRK but were less consistent in implementing the recommendations, especially among younger participants, illustrating a pronounced knowledge–practice gap. This pattern is consistent with recent data, which identified subgroups of adults combining low nutrition-related knowledge with poorer diet quality, less favourable socio-demographic profiles and inadequate physical activity. This aligns with findings from Polish adults demonstrating low nutrition-related knowledge, poorer diet quality, and less favourable socio-demographic profiles [25,34,46]. Our study is also consistent with broader methodological research showing that cluster analysis and related approaches can reveal important dietary and lifestyle patterns that are not apparent when examining single variables [47]. Our results therefore indicate that grouping NRK and NRPs provides a useful means of identifying women who may be at higher risk.

Interestingly, socio-demographic, lifestyle, and health-related factors were strongly related to both cluster membership and the distribution of NRK and NRPs in our study. Women with higher education, employment, and a better self-rated financial status were more likely to belong to the most advantageous cluster and to report greater awareness and implementation of dietary recommendations. These findings are consistent with Polish studies showing that nutrition-related knowledge and diet quality are positively associated with educational level, and that women with higher education are more likely to read food labels and engage in healthier eating habits [35]. Our results are also consistent with other evidence indicating that lower education and income, as well as unemployment and social instability, are linked to poorer diet quality, lower fruit and vegetable consumption, and higher consumption of ultra-processed foods [45].

In our study, women in the least favourable cluster combined low NRK and NRPs with less positive lifestyle indicators (more frequent smoking and lower levels of physical activity) and less favourable BMI profiles. This pattern is consistent with Polish evidence linking poorer diet, lower nutrition-related knowledge, and adverse health outcomes, including an increased risk of cardiometabolic disease [48] and breast cancer [49]. We also found that insufficient implementation of dietary guideline recommendations was more common among women with low levels of physical activity and a significant prevalence of overweight and obesity (22.4% and 10.0% of women, respectively). These findings are consistent with the lifestyle profile observed among women in Poland, where unfavourable dietary patterns frequently coexist with low levels of physical activity and elevated BMI values [50]. Although these percentages are slightly lower than recent national results for Polish women, where excess body weight and abdominal obesity are more common, particularly in middle-aged and older adults [6], the pattern we observed, in which poorer diet quality and low physical activity coexist with a higher BMI, is consistent with other Polish data [51].

Studies among Polish women have shown that lower levels of leisure-time physical activity and less healthy dietary habits are associated with less favourable BMI categories and a lifestyle profile characterized by increased cardiometabolic risk [50]. Other studies conducted in high-income populations indicate that individuals with moderate to high levels of physical activity are more likely to adhere to dietary recommendations, including consuming more fruit, vegetables, and dairy products, and tend to have a more favourable cardiometabolic profile than less active individuals [52].

### 4.1. Implications and Applications

The implications of our findings for public health practice are significant. First, our results confirm that disseminating national dietary guidelines and improving NRK are necessary but not sufficient conditions for improving NRPs. Our findings support the view that diet and physical activity should be considered together in public health strategies, rather than as separate goals [53].

Recent research suggests that interventions based on behavioural theories and focused on attitudes, perceived barriers, self-efficacy, and social influences may enhance the impact of nutrition education on actual dietary practices [14,54], and are likely to be more effective when they concurrently promote regular physical activity [24].

The results of our cluster analysis support the need for tailored strategies rather than a one-size-fits-all approach. For women in the “High” cluster (38% of the participants), maintaining and reinforcing current behaviours through ongoing access to reliable information may be sufficient. For women in the “Moderate” cluster (39%), where knowledge is not effectively translated into practice, interventions may need to prioritize behavioural skills and time- and cost-friendly solutions that reduce the gap between intention and behaviour. Of particular concern is the “Low” cluster (23%), for whom more intensive and integrated interventions are likely to be necessary, combining physical activity and nutrition education with social support and improved access to healthy foods.

In line with this evidence, we propose that interventions aimed at improving NRPs among women should go beyond information provision and include components that: (i) develop practical skills (e.g., low-cost strategies for increasing vegetable and fish intake, meal planning, healthy snacking, and incorporating simple forms of physical activity into daily routines), (ii) address environmental and financial barriers (such as access to and affordability of healthier products), and (iii) support behaviour change through psychosocial processes, such as social support, enhanced self-efficacy, and motivation building.

To better ground these recommendations, we also propose that research be based on the behavioral science framework of the COM-B model (Capability-Opportunity-Motivation-Behaviour) and the Health Action Process Approach (HAPA). The knowledge–practice gap can be grounded in the COM-B framework: NRK (knowledge of dietary guidelines) primarily reflects psychological capability (C), whereas NRPs (putting dietary guidelines into practice) represents behaviour (B) that additionally depends on opportunity (O; e.g., availability/affordability and social context) and motivation (M; processes such as intentions, and self-regulation) [55]. Consistent with HAPA, this interventions should emphasize action planning (when/where/how), coping planning (how to handle barriers such as fatigue, social occasions, or limited time), and action control (self-monitoring/feedback), rather than information alone, rather than relying on education alone [56]. We propose that future research should utilize longitudinal and intervention studies to elucidate causal pathways and test targeted strategies for specific groups of women.

### 4.2. Strengths and Limitations of the Study

Our study has several strengths. To our knowledge, no previous studies have been conducted on the impact of knowledge of national dietary guidelines on their implementation using cluster analysis identified distinct risk profiles among different age categories of adult women in Poland. Our study aimed to fill this gap and the results presented should be considered as a contribution to the data on nutrition-related knowledge and its implementation in dietary guidelines as nutrition-related practice. The significant advantage of the study lies in its large sample size (*n* = 1294) and diversity of age groups of adult women. This study identifies “Low”, “Moderate”, and “High” clusters in this population and, overall, explores the relationship between general NRK and NRPs and the corresponding socio-demographic, lifestyle, and health-related factors. Furthermore, this study identified areas of nutritional knowledge and dietary guidelines that require improvement, which could be valuable for developing personalized nutrition education programs in Poland.

However, this study also has some limitations to consider. The cross-sectional study was conducted using the CAWI method. The respondents completed the questionnaire anonymously, at a time of their choosing. Respondents were primarily women with internet skills, leading to a relatively large proportion of young women (aged 18–35 years) in the sample, which may limit the external validity and generalisability of the identified profiles to older female populations. Statistical data shows that the younger section of the population uses the internet more often than older adults [57], meaning our group is not entirely representative of the overall female population in Poland. Another limitation regarding the recruitment of older adults relates to the more frequent occurrence of health problems, social and cultural barriers, and potentially impaired capacity to provide informed consent [58]. A further limitation was the use of equal weights for all items in the NRK and NRP scales, which was intended to maintain transparency and to mitigate arbitrary or sample-specific weighting schemes that could reduce comparability with national dietary recommendations. This approach may, however, underestimate the relative importance of some items.

Regional variation was not considered in this study. Future research should aim for a more balanced distribution of women across socio-demographic parameters. Additionally, anthropometric data were obtained on the basis of self-reported data from respondents (height, body weight), which may be affected by systematic reporting bias [59]. Furthermore, this study has a cross-sectional design, and causal relationships cannot be determined from its findings.

## 5. Conclusions

We found that, in this sample of women, the self-reported awareness of national dietary guidelines was linked to higher nutrition-related knowledge (NRK) and more favourable nutrition-related practices (NRPs), indicating that guideline awareness is an important but not sufficient condition for healthy dietary behaviour. Despite generally good NRK, we found a clear knowledge–practice gap among women, particularly for recommendations regarding milk and dairy intake, regarding vegetable and fish consumption, and for maintaining adequate levels of physical activity.

Cluster analysis identified distinct profiles, with those in the “High” NRK/NRP cluster (38% of participants) demonstrating more advantageous socio-demographic, lifestyle, and health-related characteristics, while women in the “Low” cluster (23%) combined the poorest NRK and NRPs with less favourable financial, educational, and health profiles, thereby constituting a potential high-risk group for diet-related disorders.

Our findings highlight the need for multi-component, targeted public health strategies for women that go beyond information provision and combine nutrition education with practical skills, support for behaviour change, and promotion of regular physical activity, particularly among clusters with lower levels of NRK/NRP.

Future research in female populations should focus on longitudinal and intervention studies to clarify causal pathways between guideline awareness, NRK, NRPs, lifestyle factors, and health outcomes and to test tailored approaches for closing the gap between nutritional knowledge and its implementation in practice.

## Figures and Tables

**Table 1:** Women’s responses to questions assessing nutrition-related knowledge (NRK) according to self-reported knowledge of national dietary guidelines (NDGs); *n* = 1294.

Nutrition-Related Knowledge (NRK) (Questions—19 Items)	Total *n* = 1294	Knowledge of NDGs ^1^		*p*-Value *
		Yes *n* = 1082	No *n* = 212	
	*n (%) of correct answers*			
How many meals a day should be eaten?	1141 (88.2)	989 (91.4)	152 (71.7)	<0.0001
How long should the breaks between meals be?	1185 (91.6)	1004 (92.8)	181 (85.4)	0.0004
How many hours before bed should the last meal be eaten?	420 (32.5)	361 (33.4)	59 (27.8)	0.11557
How much fruit and vegetables should be eaten every day?	594 (45.9)	545 (50.4)	49 (23.1)	<0.0001
What should be the proportion of fruit and vegetable intake in the diet?	881 (68.1)	781 (72.2)	100 (47.2)	<0.0001
Which cereal products contain the most dietary fibre?	1089 (84.2)	956 (88.4)	133 (62.7)	<0.0001
How many glasses of milk should be drunk every day? (can be replaced with yoghurt, kefir, and partly cheese)	323 (25.0)	287 (26.5)	36 (17.0)	0.0033
What is the recommended amount of meat to eat in a week? (especially red meat and processed meat products)	525 (40.6)	461 (42.6)	64 (30.2)	0.0008
Which products are the best source of *n*-3 polyunsaturated fatty acids?	968 (74.8)	860 (79.5)	108 (50.9)	<0.0001
What foods should be eaten several times a week as a significant source of protein or fats?	505 (39.0)	449 (41.5)	56 (26.4)	<0.0001
What plant fats can best replace animal fats in the diet?	1089 (84.2)	938 (86.7)	151 (71.2)	<0.0001
Which fatty acids should be kept to a minimum in the diet?	749 (57.9)	673 (62.2)	76 (35.9)	<0.0001
Which cooking method should be limited?	1253 (96.8)	1057 (97.7)	196 (92.5)	<0.0001
What products can best replace sweets as snacks?	1192 (92.1)	1017 (94.0)	175 (82.6)	<0.0001
Which foods are a source of salt in the diet?	1210 (93.5)	1036 (95.8)	174 (82.1)	<0.0001
How much water should be drunk every day?	804 (62.1)	684 (63.3)	120 (56.6)	0.06952
Should the consumption of sugar-sweetened carbonated and non-carbonated beverages be limited?	1228 (94.9)	1043 (96.4)	185 (87.3)	<0.0001
What is the recommended daily alcohol intake?	955 (73.8)	821 (75.9)	134 (63.2)	<0.0001
What is at the bottom of the national dietary recommendations (Pyramid of Healthy Eating and Physical Activity/Plate of Healthy Eating)?	622 (48.1)	594 (54.9)	28 (13.2)	<0.0001

Data are *n* (%) participants (calculated within each variable). * chi-square test. ^1^ Self-reported knowledge of the Food Pyramid and/or the Healthy Eating Plate (National dietary guidelines—NDGs); answers were divided into dichotomous responses—Yes or No.

**Table 2:** Women’s responses to questions assessing nutrition-related practices (NRPs) according to self-reported knowledge of national dietary guidelines (NDGs); *n* = 1294.

Nutrition-Related Knowledge (NRK) (Questions—19 Items)	Total *n* = 1294	Knowledge of NDGs ^1^		*p*-Value *
		Yes *n* = 1082	No *n* = 212	
	*n (%) of correct answers*			
How many meals a day do you usually eat?	651 (51.9)	599 (55.4)	72 (34.0)	<0.0001
How long are the breaks between the meals you eat?	1009 (78.0)	858 (79.3)	151 (71.2)	0.0038
How many hours before bed do you usually eat your last meal?	482 (37.2)	403 (37.2)	79 (37.3)	0.9960
How many servings of fruit do you usually eat per day?	1014 (78.4)	860 (79.5)	154 (72.6)	0.0306
How many servings of vegetables do you usually eat per day?	130 (10.0)	114 (10.5)	16 (7.6)	0.1856
What cereal products do you eat most often?	517 (40.0)	467 (43.2)	50 (23.6)	<0.0001
How often do you consume milk or milk products?	182 (14.1)	159 (14.7)	23 (10.8)	0.1408
What kind of meat and/or meat products do you eat most often?	909 (70.2)	783 (72.4)	126 (59.4)	0.0002
How often do you eat fish?	94 (7.3)	81 (7.5)	13 (6.1)	0.4873
How often do you eat eggs?	593 (45.8)	505 (46.5)	90 (42.4)	0.2809
What kind of fat do you usually use for frying?	916 (70.8)	768 (71.0)	148 (69.8)	0.7323
How often do you eat fast food (fries, hamburgers, hot dogs)?	216 (16.7)	172 (15.9)	44 (20.7)	0.0828
What cooking method do you prefer most often?	386 (29.8)	326 (30.1)	60 (28.3)	0.5948
What snacks do you choose between meals most often?	488 (7.7)	393 (36.3)	95 (44.8)	0.0197
Do you add salt to food at meals?	612 (7.3)	524 (48.4)	88 (41.5)	0.0650
How much water do you drink per day?	438 (33.8)	371 (34.3)	67 (31.6)	0.4500
How often do you consume sugar-sweetened carbonated or non-carbonated beverages?	314 (24.3)	278 (25.7)	36 (17.0)	0.0068
How often do you drink alcohol?	254 (19.6)	210 (19.4)	44 (20.8)	0.6518
How do you rate your physical activity?	121 (9.4)	98 (9.1)	23 (10.9)	0.4126

Data are *n* (%) participants (calculated within each variable). * chi-square test. ^1^ self-reported knowledge of the Food Pyramid and/or the Healthy Eating Plate (national dietary guidelines (NDGs)); answers were divided into dichotomous responses—Yes or No.

**Table 3:** Assessment of nutrition-related knowledge (NRK) and nutrition-related practices (NRPs) in relation to self-reported knowledge of national dietary guidelines (NDGs); *n* = 1294.

Variables	Total *n* = 1294		Knowledge of NDGs ^1^				*p*-Value *
			Yes *n* = 1082		No *n* = 212		
	Median	IQR	Median	IQR	Median	IQR	
NRK score	13.0	11–15	14.0	12–15	10.0	8–12	<0.0001 *^1^
NRP score	9.0	7–11	9.0	7–11	7.0	6–10	<0.0001 *^1^
NRK vs. NRP	<0.0001 *^2^		<0.0001 *^2^		<0.0001 *^2^		
Low (≤5 points)	19 (1.5)		5 (0.5)		14 (6.6)		<0.0001 *^3^
Moderate (6–10 points)	238 (18.4)		120 (11.1)		118 (55.7)		
High (11–15 points)	721 (55.7)		644 (59.5)		77 (36.3)		
Very high (≥16 points)	316 (24.4)		313 (28.9)		3 (1.4)		
Low (≤5 points)	79 (6.1)		58 (5.4)		21 (9.4)		<0.0001 *^3^
Moderate (6–10 points)	798 (61.7)		644 (59.5)		154 (74.5)		
High (11–15 points)	398 (30.7)		365 (33.7)		33 (15.6)		
Very high (≥16 points)	19 (1.5)		15 (1.4)		4 (0.5)		

Data are *n* (%) participants (calculated within each variable). Level of nutrition-related knowledge (NRK) and nutrition-related practices (NRPs): ≤5 points—low; 6–10 points—moderate; 11–15 points—high; ≥16 points—very high; total scores (possible range: 0–19). * p-value: *^1^ Kruskal–Wallis rank test; *^2^ Wilcoxon signed-rank test; *^3^ chi-square test; ^1^ self-reported indicator was applied to assess knowledge of the Food Pyramid and/or the Healthy Eating Plate (National dietary guidelines—NDGs); answers were divided into dichotomous Yes or No.

**Table 4:** Description of NRK and NRP scores; age and BMI values by clusters corresponding to “Low”, “Moderate”, and “High” knowledge of national dietary guidelines; *n* = 1294.

Variables	Clusters ^1^			Effect Size	*p*-Value *
	Low	Moderate	High		
	*n* = 299	*n* = 503	*n* = 492		
	Median (range)/IQR				
NRK score (points) ^2^	11.0 ^a^ (2–16) 9–13	13.0 ^b^ (2–17) 11–14	15.0 ^c^ (10–19) 14–17	0.396 ^†^	<0.0001 *^1^
	Median (range)/IQR				
NRP score (points) ^2^	8.0 ^a^ (3–16) 7–10	7.0 ^b^ (3–13) 6–9	11.0 ^c^ (7–18) 10–13	0.435 ^†^	<0.0001 *^2^
	Median (range)/IQR				
Age (years)	56.0 ^a^ (22–80) 45–64	25.0 ^b^ (18–57) 23–32	30.0 ^c^ (18–71) 24–40	0.498 ^†^	<0.0001 *^1^
Age groups:	*n* (%) participants				
18–25 y	8 (2.7)	258 (51.3)	182 (37.0)		
26–35 y	24 (8.0)	158 (31.4)	127 (25.8)	0.504 ^††^	<0.0001 *^2^
36–45 y	63 (21.1)	84 (16.7)	134 (27.2)		
>45 y	204 (68.2)	3 (0.6)	49 (10.0)		
	Median (range)/IQR				
BMI (kg/m^2^)	27.9 ^a^ (17.9–47.8) 25.0–30.8	21.1 ^b^ (15.4–31.2) 19.9–23.5	22.5 ^b^ (16.5–38.6) 20.2–24.8	0.315 ^†^	<0.0001 *^1^
Weight status:	*n* (%) participants				
Underweight	1 (0.3)	52 (10.3)	38 (7.7)		
Normal	74 (24.8)	372 (74.0)	340 (69.1)	0.380 ^††^	<0.0001 *^2^
Overweight	131 (43.8)	68 (13.5)	91 (18.5)		
Obesity	93 (31.1)	11 (2.2)	23 (4.7)		

^1^ NRK and NRP patterns, including age and BMI, were identified using K-means cluster analysis and categorized into “Low”, “Moderate”, and “High” knowledge of national dietary guidelines. ^2^ Level of nutrition-related knowledge (NRK) and nutrition-related practice (NRP): possible range: 0–19 points. * *p*-value: *^1^ Kruskal–Wallis rank test; ^a, b, c^—different letters indicate statistically significant differences between groups, *p* < 0.05; *^2^ chi-square; Effect size: ^†^ Eta-squared (η^2^); ^††^ V-Cramér’s V coefficient (V). *n* (%) participants (calculated within each cluster).

**Table 5:** Socio-demographic, lifestyle and health-related factors by clusters; *n* = 1294.

Variables	Clusters ^1^			Effect Sizes	*p*-Value *
	Low	Moderate	High		
	*n* = 299	*n* = 503	*n* = 492		
	Place of residence				
Rural	101 (33.8)	178 (35.4)	169 (34.4)	0.236	<0.0001
Small town (<20,000 inhabitants)	61 (20.4)	37 (7.4)	40 (8.1)		
Town (20,000–100,000 inhabitants)	55 (18.4)	87 (17.3)	74 (15.0)		
City (>100,000 inhabitants)	82 (27.4)	201 (40.0)	209 (42.5)		
	Education level				
Primary	28 (9.4)	25 (5.0)	9 (1.8)	0.375	<0.0001
Vocational	68 (22.7)	9 (1.8)	14 (2.9)		
Secondary	87 (29.1)	168 (33.4)	123 (25.0)		
Higher	116 (38.8)	301 (59.8)	346 (70.3)		
	Occupation status				
Employed	110 (63.2)	154 (69.3)	139 (81.7)	0.090	0.0405
Unemployed	189 (36.8)	348 (30.7)	353 (28.3)		
	Financial status (self-perceived)				
Very bad	14 (4.7)	33 (6.6)	38 (7.7)	0.259	<0.0001
Bad	13 (4.3)	19 (3.8)	5 (1.0)		
Sufficient	162 (54.2)	215 (42.7)	168 (34.1)		
Good	109 (36.5)	229 (45.5)	276 (56.5)		
Very good	1 (0.3)	7 (1.4)	3 (0.6)		
	Health status (self-rated)				
Poor	25 (8.4)	34 (6.7)	19 (3.9)	0.286	<0.0001
Fair	162 (54.2)	189 (37.6)	133 (27.0)		
Good	112 (37.4)	208 (55.7)	340 (69.1)		
	Use of dietary supplements				
Yes	102 (34.1)	160 (31.8)	242 (49.2)	0.185	<0.0001
No	197 (65.9)	343 (68.2)	250 (50.8)		
	Following a special diet for 6 months before the study				
Yes	39 (13.0)	66 (13.1)	75 (15.2)	0.130	0.5543
No	260 (87.0)	437 (86.9)	417 (84.8)		
	Currently smoking				
Yes	82 (27.4)	128 (25.5)	67 (13.6)	0.225	<0.0001
No	217 (72.6)	374 (74.5)	424 (86.4)		
	Alcohol consumption				
Never	73 (24.5)	58 (11.6)	123 (25.1)	0.193	<0.0001
1-several times/month	165 (55.4)	336 (67.1)	287 (58.7)		
1-several times/week	59 (19.8)	104 (20.8)	78 (16.0)		
1-several times/day	1 (0.3)	3 (0.6)	1 (0.2)		
	Physical activity level (self-perceived)				
Low	159 (53.2)	227 (45.1)	170 (34.5)	0.185	<0.0001
Moderate	110 (36.8)	221 (43.9)	235 (47.7)		
High	30 (10.0)	55 (10.9)	87 (17.7)		

^1^ NRK and NRP patterns, including age and BMI, identified using K-means cluster analysis and categorized into “Low”, “Moderate”, and “High” knowledge of national dietary guidelines. * *p*-value: chi-square test; effect size: V-Cramér’s V coefficient (V). *n* (%) participants (calculated within each cluster).

## Data Availability

The data presented in this study are available on request from the corresponding author upon reasonable request.

## Supplementary Materials

The following supporting information can be downloaded at: https://www.mdpi.com/article/10.3390/nu17243916/s1, Table S1: List of the questionnaire items measuring nutrition-related knowledge (NRK) reflected in the national dietary guidelines for adults with rewarded response; Table S2: List of the questionnaire items measuring nutrition-related practice (NRP) reflected in the national dietary guidelines for adults with rewarded response. Table S3. Socio-demographic, lifestyle, and health-related characteristics of the study population (*n* = 1294). Figure S1. Elbow plot representing the number of clusters in the k-means algorithm (*n* = 1294).

## References

[1] Tafuri D., Latino F. (2025). Association of Dietary Intake with Chronic Disease and Human Health. Nutrients.

[2] Santos L. (2022). The Impact of Nutrition and Lifestyle Modification on Health. Eur. J. Intern. Med..

[3] Chew N.W.S., Ng C.H., Tan D.J.H., Kong G., Lin C., Chin Y.H., Lim W.H., Huang D.Q., Quek J., Fu C.E. (2023). The Global Burden of Metabolic Disease: Data from 2000 to 2019. Cell Metab..

[4] Powell-Wiley T.M., Poirier P., Burke L.E., Després J.-P., Gordon-Larsen P., Lavie C.J., Lear S.A., Ndumele C.E., Neeland I.J., Sanders P. (2021). Obesity and Cardiovascular Disease: A Scientific Statement From the American Heart Association. Circulation.

[5] Zhou X.-D., Chen Q.-F., Yang W., Zuluaga M., Targher G., Byrne C.D., Valenti L., Luo F., Katsouras C.S., Thaher O. (2024). Burden of Disease Attributable to High Body Mass Index: An Analysis of Data from the Global Burden of Disease Study 2021. eClinicalMedicine.

[6] Traczyk I., Kucharska A., Sińska B.I., Panczyk M., Samel-Kowalik P., Kłak A., Raciborski F., Wyleżoł M., Samoliński B., Szostak-Węgierek D. (2024). Prevalence of Overweight, Obesity, and Abdominal Obesity in Polish Adults: Sociodemographic Analysis from the 2016–2020 National Health Program. Nutrients.

[7] Stoś K., Rychlik E., Woźniak A., Ołtarzewski M., Wojda B., Przygoda B., Matczuk E., Pietraś E., Kłys W. (2021). Krajowe Badanie Sposobu Żywienia i Stanu Odżywienia Populacji Polskiej. Narodowy Instytut Zdrowia Publicznego PZH—Państwowy Instytut Badawczy. Warszawa. https://ncez.pzh.gov.pl/wp-content/uploads/2021/10/Raport-z-projektu-EFSA-18.10.pdf.

[8] Jezewska-Zychowicz M., Wadolowska L., Kowalkowska J., Lonnie M., Czarnocinska J., Babicz-Zielinska E. (2017). Perceived Health and Nutrition Concerns as Predictors of Dietary Patterns among Polish Females Aged 13–21 Years (GEBaHealth Project). Nutrients.

[9] Białek-Dratwa A., Kokot T., Czech E., Całyniuk B., Kiciak A., Staśkiewicz W., Stanjek-Cichoracka A., Słoma-Krześlak M., Sobek O., Kujawińska M. (2023). Dietary Trends among Polish Women in 2011–2022-Cross-Sectional Study of Food Consumption Frequency among Women Aged 20–50 in Silesia Region, Poland. Front. Nutr..

[10] Bolesławska I., Błaszczyk-Bębenek E., Jagielski P., Jagielska A., Przysławski J. (2021). Nutritional Behaviors of Women and Men in Poland during Confinement Related to the SARS-CoV-2 Epidemic. Sci. Rep..

[11] Dardzińska J.A., Małgorzewicz S., Szupryczyńska N., Gładyś K., Śliwińska A., Kaczkan M., Pieszko M., Wojda A., Wernio E., Gogga P. (2023). Adherence to the 2021 Dietary Guidelines of the European Society of Cardiology on Cardiovascular Disease Prevention in Residents of the Pomeranian Voivodeship with Increased Cardiovascular Risk. Pol. Arch. Intern. Med..

[12] Stewart T.M., Martin C.K., Williamson D.A. (2022). The Complicated Relationship between Dieting, Dietary Restraint, Caloric Restriction, and Eating Disorders: Is a Shift in Public Health Messaging Warranted?. Int. J. Environ. Res. Public Health.

[13] Zaiser C., Pahlenkemper M., Brandt G., Ballero Reque C., Sabel L., Laskowski N.M., Paslakis G. (2025). Feeding the Feelings: Gender Differences in Emotional Eating during COVID-19: A Systematic Review and Meta-Analysis. Front. Nutr..

[14] Bolognese M.A., Franco C.B., Ferrari A., Bennemann R.M., Lopes S.M.A., Bertolini S.M.M.G., Júnior N.N., Branco B.H.M. (2020). Group Nutrition Counseling or Individualized Prescription for Women With Obesity? A Clinical Trial. Front. Public Health.

[15] Marías Y.F., Glasauer P. (2014). Guidelines for Assessing Nutrition-Related Knowledge, Attitudes and Practices.

[16] Murimi M.W., Kanyi M., Mupfudze T., Amin M.R., Mbogori T., Aldubayan K. (2017). Factors Influencing Efficacy of Nutrition Education Interventions: A Systematic Review. J. Nutr. Educ. Behav..

[17] Sigala E.G., Pitsavos C., Barkas F., Liberopoulos E., Sfikakis P.P., Tsioufis C., Panagiotakos D. (2025). Sex-Based Associations Between Education Level, EAT–Lancet Diet, and 20-Year Cardiovascular Risk: The ATTICA Study (2002–2022). Nutrients.

[18] Pavicic E., Vincenz A., Bitterlich N., Von Wolff M., Stute P. (2024). Women’s Self-Concept of and Real Knowledge about Nutrition: A Cross-Sectional Study. Womens Health.

[19] Aureli V., Rossi L. (2022). Nutrition Knowledge as a Driver of Adherence to the Mediterranean Diet in Italy. Front. Nutr..

[20] Jeruszka-Bielak M., Kollajtis-Dolowy A., Santoro A., Ostan R., Berendsen A.A.M., Jennings A., Meunier N., Marseglia A., Caumon E., Gillings R. (2018). Are Nutrition-Related Knowledge and Attitudes Reflected in Lifestyle and Health Among Elderly People? A Study Across Five European Countries. Front. Physiol..

[21] Boujelbane M.A., Ammar A., Salem A., Kerkeni M., Trabelsi K., Bouaziz B., Masmoudi L., Heydenreich J., Schallhorn C., Müller G. (2025). Gender-Specific Insights into Adherence to Mediterranean Diet and Lifestyle: Analysis of 4,000 Responses from the MEDIET4ALL Project. Front. Nutr..

[22] Glick A.A., Winham D.M., Heer M.M., Hutchins A.M., Shelley M.C. (2025). Nutrition Knowledge Varies by Food Group and Nutrient Among Adults. Foods.

[23] Pinho M.G.M., Mackenbach J.D., Charreire H., Oppert J.-M., Bárdos H., Glonti K., Rutter H., Compernolle S., De Bourdeaudhuij I., Beulens J.W.J. (2018). Exploring the Relationship between Perceived Barriers to Healthy Eating and Dietary Behaviours in European Adults. Eur. J. Nutr..

[24] Lee J., Walker M.E., Bourdillon M.T., Spartano N.L., Rogers G.T., Jacques P.F., Vasan R.S., Xanthakis V. (2021). Conjoint Associations of Adherence to Physical Activity and Dietary Guidelines With Cardiometabolic Health: The Framingham Heart Study. J. Am. Heart Assoc..

[25] Kucharska A., Sińska B.I., Panczyk M., Samel-Kowalik P., Raciborski F., Czerwonogrodzka-Senczyna A., Boniecka I., Traczyk I. (2025). Nutritional Knowledge, Sociodemographic, and Lifestyle Factors as Determinants of Diet Quality—A Polish Population-Based Study. Front. Public Health.

[26] Leme A.C.B., Hou S., Fisberg R.M., Fisberg M., Haines J. (2021). Adherence to Food-Based Dietary Guidelines: A Systemic Review of High-Income and Low- and Middle-Income Countries. Nutrients.

[27] Bechthold A., Boeing H., Tetens I., Schwingshackl L., Nöthlings U. (2018). Perspective: Food-Based Dietary Guidelines in Europe-Scientific Concepts, Current Status, and Perspectives. Adv. Nutr..

[28] *Pyramid of Healthy Nutrition and Physical Activity for Adults*; National Center for Nutrition Education; National Food and Nutrition Institute: Warsaw, Poland, 2018. https://ncez.pzh.gov.pl/abc-zywienia/zasady-zdrowego-zywienia/piramida-zdrowego-zywienia-i-aktywnosci-fizycznej-dla-osob-doroslych-2/.

[29] (2020). *Healthy Eating Plate*; National Center for Nutrition Education; National Institute of Public Health NIH—National Research Institute: Warsaw, Poland. https://ncez.pzh.gov.pl/abc-zywienia/talerz-zdrowego-zywienia/.

[30] Wawrzyniak A., Traczyk I. (2024). Nutrition-Related Knowledge and Nutrition-Related Practice among Polish Adolescents—A Cross-Sectional Study. Nutrients.

[31] Wang X., Ji X. (2020). Sample Size Estimation in Clinical Research. Chest.

[32] Jeżewska-Zychowicz M., Gawęcki J., Wądołowska L., Czarnocińska J., Galiński G., Kołłajtis-Dołowy A., Roszkowski W., Wawrzyniak A., Przybyłowicz K., Stasiewicz B., Gawecki J. (2024). KomPAN^®^ Dietary Habits and Nutrition Beliefs Questionnaire or Adolescents Aged 16–18 Years and Adults Version 2.2.—Self-Administered Questionnaire. KomPAN^®^ Dietary Habits and Nutrition Beliefs Questionnaire and the Manual for Developing of Nutritional Data.

[33] WHO Consultation (2000). Obesity: Preventing and Managing the Global Epidemic. Report of a WHO Consultation. World Health Organ. Tech. Rep. Ser..

[34] Jezewska-Zychowicz M., Plichta M. (2022). Diet Quality, Dieting, Attitudes and Nutrition Knowledge: Their Relationship in Polish Young Adults—A Cross-Sectional Study. Int. J. Environ. Res. Public Health.

[35] Żarnowski A., Jankowski M., Gujski M. (2022). Nutrition Knowledge, Dietary Habits, and Food Labels Use—A Representative Cross-Sectional Survey among Adults in Poland. Int. J. Environ. Res. Public Health.

[36] Spronk I., Kullen C., Burdon C., O’Connor H. (2014). Relationship between Nutrition Knowledge and Dietary Intake. Br. J. Nutr..

[37] Kotowska A., Sochacka K., Wiśniewski R., Lachowicz-Wiśniewska S. (2024). Dietary Habits of Young Poles and Their Selected Determinants: A Review and Implications for Public Health. Nutrients.

[38] De Vriendt T., Matthys C., Verbeke W., Pynaert I., De Henauw S. (2009). Determinants of Nutrition Knowledge in Young and Middle-Aged Belgian Women and the Association with Their Dietary Behaviour. Appetite.

[39] Scalvedi M.L., Gennaro L., Saba A., Rossi L. (2021). Relationship Between Nutrition Knowledge and Dietary Intake: An Assessment Among a Sample of Italian Adults. Front. Nutr..

[40] Jakubowska D., Dąbrowska A.Z., Staniewska K., Kiełczewska K., Przybyłowicz K.E., Żulewska J., Łobacz A. (2024). Health Benefits of Dairy Products’ Consumption—Consumer Point of View. Foods.

[41] Comerford K.B., Miller G.D., Boileau A.C., Masiello Schuette S.N., Giddens J.C., Brown K.A. (2021). Global Review of Dairy Recommendations in Food-Based Dietary Guidelines. Front. Nutr..

[42] Bréjon M., Tavares F., Florença S.G., Gonçalves J.C., Barroca M.J., Guiné R.P.F. (2024). Knowledge about Consumption of Milk: Study Involving Consumers from Two European Countries—France and Portugal. Open Agric..

[43] Craig W.J., Messina V., Rowland I., Frankowska A., Bradbury J., Smetana S., Medici E. (2023). Plant-Based Dairy Alternatives Contribute to a Healthy and Sustainable Diet. Nutrients.

[44] Miller V., Webb P., Cudhea F., Shi P., Zhang J., Reedy J., Erndt-Marino J., Coates J., Mozaffarian D., Global Dietary Database (2022). Global Dietary Quality in 185 Countries from 1990 to 2018 Show Wide Differences by Nation, Age, Education, and Urbanicity. Nat. Food.

[45] Klink U., Intemann T., Bogl L.H., Lissner L., Gwozdz W., De Henauw S., Molnár D., Mazur A., Moreno L.A., Pala V. (2025). Consumer Attitudes towards Dietary Behaviors: A Mediator between Socioeconomic Status and Diet Quality in European Adults. Eur. J. Nutr..

[46] Koch F., Hoffmann I., Claupein E. (2021). Types of Nutrition Knowledge, Their Socio-Demographic Determinants and Their Association With Food Consumption: Results of the NEMONIT Study. Front. Nutr..

[47] Devlin U.M., McNulty B.A., Nugent A.P., Gibney M.J. (2012). The Use of Cluster Analysis to Derive Dietary Patterns: Methodological Considerations, Reproducibility, Validity and the Effect of Energy Mis-Reporting. Proc. Nutr. Soc..

[48] Waśkiewicz A., Szcześniewska D., Szostak-Węgierek D., Kwaśniewska M., Pająk A., Stepaniak U., Kozakiewicz K., Tykarski A., Zdrojewski T., Zujko M.E. (2016). Are Dietary Habits of the Polish Population Consistent with the Recommendations for Prevention of Cardiovascular Disease?—WOBASZ II Project. Kardiol. Pol..

[49] Stasiewicz B., Biernacki M., Slowinska M.A., Wadolowska L. (2025). Associations of Nutritional Knowledge with Dietary Patterns and Breast Cancer Occurrence. Sci. Rep..

[50] Ostachowska-Gasior A., Kolarzyk E., Majewska R., Gasior A., Kwiatkowski J., Zaleska I. (2018). Diet and Physical Activity as Determinants of Lifestyle Chosen by Women from Southern Poland. Int. J. Environ. Res. Public Health.

[51] Sierpiński R., Jankowski M., Raciborski F. (2025). Differences in Lifestyle-Related Behaviors Among Healthy Weight, Overweight, and Obese Groups: A Secondary Analysis of Data on 4714 Adults in Poland. Nutrients.

[52] Wang W., Zhou H., Qi S., Yang H., Hong X. (2024). The Association between Physical Activities Combined with Dietary Habits and Cardiovascular Risk Factors. Heliyon.

[53] Mihalache O.A., Nicolau A.I., Elliott C., Dall’Asta C. (2025). Food and Health Professionals and Aspirant Professionals’ Knowledge, Attitude, and Practices Related to the Risks and Benefits of Dietary Patterns: A Structural Equation Modelling Approach in Three European Countries. Future Foods.

[54] Feskens E.J.M., Bailey R., Bhutta Z., Biesalski H.-K., Eicher-Miller H., Krämer K., Pan W.-H., Griffiths J.C. (2022). Women’s Health: Optimal Nutrition throughout the Lifecycle. Eur. J. Nutr..

[55] Carey S., Hogan S. (2025). Using the Theoretical Domains Framework and Behavior Change Wheel Framework within the World of Nutrition Support. Nutr. Clin. Pract..

[56] Kettler C., Weber R.-M., Anand C., Husain S., Koeder C., Schoch N., Michaelsen M.M., Esch T., Englert H. (2025). Effect of a Community-Based Lifestyle Intervention on Predictors of Behavior Change Regarding a Healthy Plant-Based Diet—The Healthy Lifestyle Community Program (Cohort 2). Front. Public Health.

[57] GUS Wykorzystanie Technologii Informacyjno-Komunikacyjnych w Jednostkach Administracji Publicznej, Przedsiębiorstwach i Gospodarstwach Domowych w 2022 Roku. https://stat.gov.pl/obszary-tematyczne/nauka-i-technika-spoleczenstwo-informacyjne/spoleczenstwo-informacyjne/wykorzystanie-technologii-informacyjno-komunikacyjnych-w-jednostkach-administracji-publicznej-przedsiebiorstwach-i-gospodarstwach-domowych-w-2022-roku,3,21.html.

[58] Pineda E., Poelman M.P., Aaspõllu A., Bica M., Bouzas C., Carrano E., De Miguel-Etayo P., Djojosoeparto S., Blenkuš M.G., Graca P. (2022). Policy Implementation and Priorities to Create Healthy Food Environments Using the Healthy Food Environment Policy Index (Food-EPI): A Pooled Level Analysis across Eleven European Countries. Lancet Reg. Health Eur..

[59] Hattori A., Sturm R. (2013). The Obesity Epidemic and Changes in Self-report Biases in BMI. Obesity.

