# Supplementary material for: Identifying Distinct Profiles of Nutrition Knowledge and Dietary Practices, and Their Determinants Among Adult Women: A Cross-Sectional Study
Source: Nutrients. 2025 Dec 14;17(24):3916. doi: 10.3390/nu17243916 (PMC12735520; doi:10.3390/nu17243916)
Supplement: Supplementary file 1 [file nutrients-17-03916-s001.zip › Figure S1.pdf]

Figure S1. Elbow plot representing the number of clusters in k-means algorithm (n = 1,294).

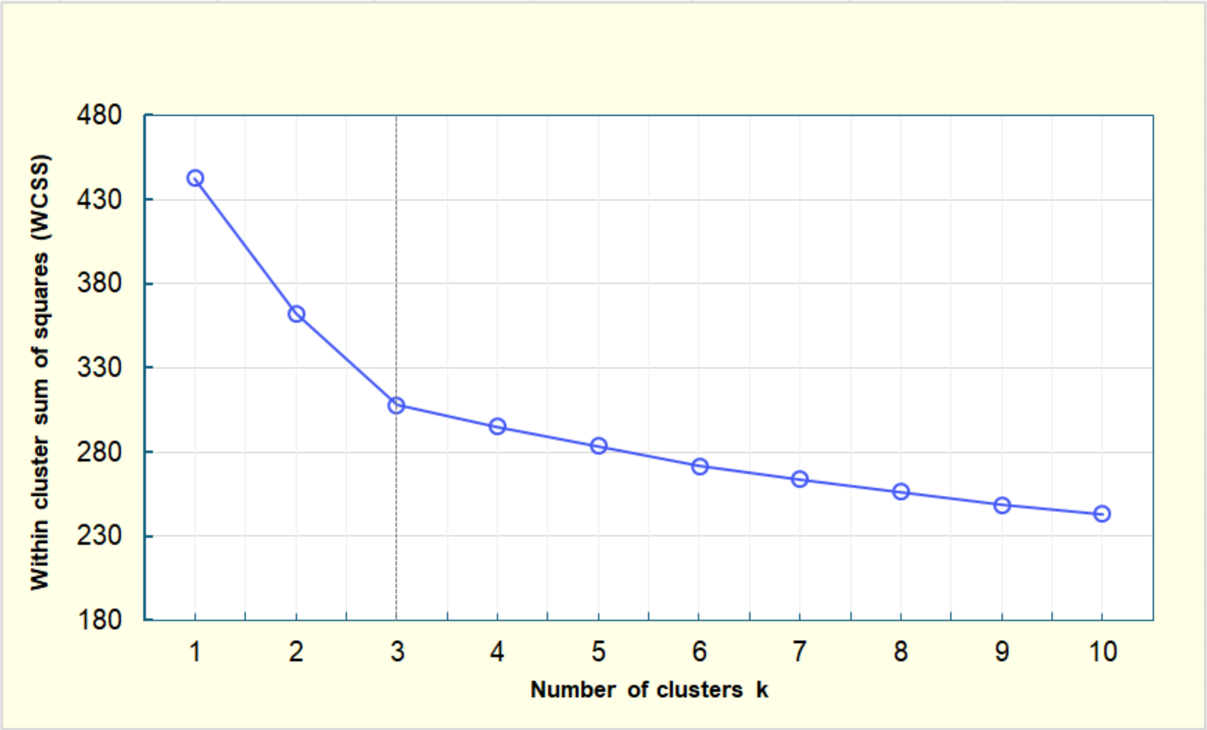

The Elbow plot was used to determine the number of clusters in the k-means algorithm. Before applying clustering analysis, the data were transformed into z-scores to standardise variables with different units and ranges. The within-cluster sum of squares (WCSS) values, which measure the total distance between each data point and its assigned cluster centroid within each cluster, were plotted to create an elbow plot, where the elbow point was defined as the optimal number of clusters.
