# Supplementary material for: Identifying Distinct Profiles of Nutrition Knowledge and Dietary Practices, and Their Determinants Among Adult Women: A Cross-Sectional Study
Source: Nutrients. 2025 Dec 14;17(24):3916. doi: 10.3390/nu17243916 (PMC12735520; doi:10.3390/nu17243916)
Supplement: Supplementary file 1 [file nutrients-17-03916-s001.zip › Table S1 and Table S2.pdf]

Supplementary

Table S1. List of the questionnaire items measuring nutrition-related knowledge (NRK) reflected to the national dietary guidelines for adults with rewarded response.

No	Questions	Awarded answer (1 point)
Q1	How many meals a day should be eaten?	4-5 meals
Q2	How long should the breaks between meals be?	3-4 hours
Q3	How many hours before bed should the last meal be eaten?	3 hours
Q4	How much fruit and vegetables should be eaten every day?	at least 400g
Q5	What should be the proportion of fruit and vegetable intake in the diet?	1/4 fruit, 3/4 vegetables
Q6	Which cereal products contain the most dietary fiber?	whole-grain cereal products, e.g. wholemeal bread, oatmeal, brown rice, wholegrain pasta
Q7	How many glasses of milk should be drunk every day? (can be replaced with yoghurt, kefir and - partly - cheese)	at least 2 serving a day
Q8	What is the recommended amount of meat to eat in a week? (especially red meat and processed meat products)	up to 0.5 kg a week
Q9	Which products are the best source of n-3 polyunsaturated fatty acids?	fatty sea fish
Q10	What foods should be eaten several times a week as a valuable source of protein or fats?	eggs
Q11	What plant fats can best replace animal fats in the diet?	rapeseed oil, olive oil
Q12	Which fatty acids should be kept to a minimum in the diet?	saturated fatty acids
Q13	Which cooking method should be limited?	frying
Q14	What products can best replace sweets as snacks?	fruit, and nuts and seeds
Q15	Which foods are a source of salt in the diet?	fast food and salty snacks
Q16	How much water should be drunk every day?	at least 1.5 litters
Q17	Should the consumption of sweetened carbonated and non-carbonated beverages be limited?	is recommended
Q18	What is the recommended daily alcohol intake?	is not recommended
Q19	What is at the bottom of the national healthy eating recommendations (Pyramid of Healthy Eating/Plate of Healthy Eating)?	physical activity

Table S2. List of the questionnaire items measuring nutrition-related practice (NRP) reflected to the national dietary guidelines for adults with rewarded response.

No	Questions	Awarded answer (1 point)
Q1	How many meals a day do you usually eat?	4-5 meals
Q2	How long are the breaks between the meals you eat?	3-4 hours
Q3	How many hours before bed do you usually eat your last meal?	3 hours
Q4	How many servings of fruit do you usually eat per day?	1-2 servings
Q5	How many servings of vegetables do you usually eat per day?	4-5 servings
Q6	What cereal products do you eat most often?	whole-grain cereal products, e.g. wholemeal bread, oatmeal, brown rice, wholegrain pasta
Q7	How often do you consume milk or milk products?	few times a day
Q8	What kind of meat and/or meat products do you eat most often?	lean meat, e.g. chicken/turkey breast, pork loin
Q9	How often do you eat fish?	At least 2 serving per week
Q10	How often do you eat eggs?	few times a week
Q11	What kind of fat do you usually use for frying?	refined rapeseed oil, olive oil
Q12	How often do you eat fast food (french fries, hamburgers, hot dogs)?	never
Q13	What cooking method do you prefer most often?	cooking, including steaming, stewing or baking
Q14	What snacks do you choose between meals most often?	vegetables, fruits, nuts, seeds, low-fat dairy products or answer I don't eat snacks
Q15	Do you add salt to food at meals?	never
Q16	How much water do you drink per day?	at least 1.5 liters
Q17	How often do you consume sweetened carbonated or non-carbonated beverages?	never
Q18	How often do you drink alcohol?	never
Q19	How do you rate your physical activity?	high – spending most of the time actively
