# Supplementary material for: Identifying Distinct Profiles of Nutrition Knowledge and Dietary Practices, and Their Determinants Among Adult Women: A Cross-Sectional Study
Source: Nutrients. 2025 Dec 14;17(24):3916. doi: 10.3390/nu17243916 (PMC12735520; doi:10.3390/nu17243916)
Supplement: Supplementary file 1 [file nutrients-17-03916-s001.zip › Table S3.pdf]

Table S3. Socio-demographic, lifestyle and health-related characteristics of the study population (n = 1,294).

Variables	n (%) participants
**Age groups**	
18-25 y	448 (34.6)
26-35 y	309 (23.9)
36-45 y	281 (21.7)
>45 y	256 (19.8)
**Place of residence**	
Rural	448 (34.6)
Small town (<20,000 inhabitants)	216 (16.7)
Town (20,000-100,000 inhabitants)	492 (38.0)
City (>100,000 inhabitants)	138 (10.7)
**Education level**	
Primary	62 (4.8)
Vocational	91 (7.0)
Secondary	378 (29.2)
Higher	763 (59.0)
**Occupation Status**	
Employed	890 (68.8)
Unemployed	403 (31.2)
**Financial status (self-perceived)**	
Very bad	10 (0.8)
Bad	37 (2.8)
Sufficient	545 (42.1)
Good	616 (47.6)
Very good	85 (6.6)
**Health status (self-rated)**	
Poor	78 (6.0)
Fair	448 (37.4)
Good	732 (56.6)
**Use of dietary supplements**	
Yes	504 (38.9)
No	790 (61.1)
**Following a special diet 6 months before the study**	
Yes	180 (13.9)
No	1114 (86.1)
**Physical activity level (self-perceived)**	
Low	556 (43.0)
Moderate	566 (43.7)
High	172 (13.3)
**Currently smoking**	
Yes	277 (21.4)
No	1016 (78.6)
**Alcohol consumption**	
Never	254 (19.6)
1-several times/month	788 (60.9)
1-several times/week	241 (18.6)
1- several times/day	5 (0.4)
**Weight status (BMI category)**	
Underweight	91 (7.0)
Normal	786 (60.7)
Overweight	280 (22.4)
Obesity	127 (9.8)

**Knowledge of national dietary guidelines (self-reported)**[1]	
Yes	1082 (83.6)
No	212 (16.4)

[1] National Dietary Guidelines (NDG) – based on the Pyramid of Healthy Eating and the Plate of Healthy Eating. Data are n (%) participants (calculated within each variables).
